# 2,4-Epibrassinolide Enhances Drought Tolerance in *Prunella vulgaris* by Improving Photosynthesis, Redox Homeostasis, and Secondary Metabolism

**DOI:** 10.3390/plants14233587

**Published:** 2025-11-25

**Authors:** Qingshan Chang, Yiming Sun, Hairui Yao, Biao Zhang, Lixia Zhang, Zi Wang, Qiaoming Zhang, Sudan Chen, Rongrong Liu, Wenxin Chang, Xiaohui Wang, Yiqi Zheng, Xiaogai Hou

**Affiliations:** 1 College of Horticulture and Plant Protection, Henan University of Science and Technology, Luoyang 471000, China; hkdcqs@126.com (Q.C.);; 2 Henan Key Laboratory of Germplasm Innovation and Utilization of Eco-Economic Woody Plant, Luoyang 471023, China; 3 College of Agriculture, Henan University of Science and Technology, Luoyang 471000, China; 4 Peony Research Institute, Luoyang Academy of Agriculture and Forestry Sciences, Luoyang 471023, China

**Keywords:** *Prunella vulgaris*, 2,4-epibrassinolide (EBR), drought stress, secondary metabolism, root system, gene expression

## Abstract

As a species of significant traditional medicinal importance, *Prunella vulgaris* is severely limited by drought stress, given its high sensitivity to this environmental constraint. 24-epibrassinolide (EBR) has shown promise in enhancing plant stress resilience and secondary metabolite production, yet its efficacy in mitigating drought effects on *P. vulgaris* requires further elucidation. In this study, foliar application of EBR (0, 0.01, 0.05, 0.1, 0.2 μmol·L^−1^) was applied to drought-stressed *P. vulgaris* seedlings (maintained at 60% ± 5% field capacity, FC, for 20 days during the flowering stage; control at 75% ± 5% FC). The results showed that drought inhibited the growth and development of *P. vulgaris*. Compared with the control group, malondialdehyde, hydrogen peroxide, and superoxide anion increased by 77.82%, 27.47%, and 44.95%, respectively. The total chlorophyll content and the coordination between photosystem I and photosystem II decreased by 42.33% and 46.62%, respectively. Additionally, the net photosynthetic rate and biomass of *P. vulgaris* significantly decreased by 45.12% and 34.66%, respectively. In contrast, the 0.1 μmol·L^−1^ EBR significantly enhanced the antioxidant and osmoregulation systems. Compared with drought stress treatment, the activities of SOD, POD, CAT, APX and GPX increased by 10.78%, 45.86%, 48.44%, 40.58% and 63.37%, respectively; soluble sugar, soluble protein and proline contents increased by 53.38%, 29.09% and 45.95%, respectively; and malondialdehyde, hydrogen peroxide and superoxide anion levels decreased by 28.37%, 15.77% and 25.73%, respectively. Total chlorophyll content, photosystem coordination and net photosynthetic rate increased by 55.68%, 43.08% and 45.88%, respectively, along with a significant 42.23% increase in total biomass. Furthermore, EBR upregulated the transcription levels of key phenylpropanoid pathway genes and elevated secondary metabolite contents. The expression of *Pv4CL*, *PvC4H*, *PvPAL* and *PvTAT* increased by 26.97%, 90.42%, 35.52% and 84.35%, respectively. Accordingly, total phenolic content, caffeic acid, ferulic acid, rosmarinic acid and hyperoside increased by 36.44%, 121.01%, 100.27%, 72.38% and 80.77%, respectively. Lower EBR concentrations (0.01 μmol·L^−1^) had no significant effect on most indices, while 0.2 μmol L^−1^ EBR showed weakened effects. In summary, under 60% ± 5% field capacity (FC) drought, 2,4-epibrassinolide (EBR) enhances drought adaptation, medicinal yield, and quality of *P. vulgaris*, with 0.1 μmol L^−1^ EBR as the optimal concentration. This improvement is driven by enhanced antioxidant capacity, optimized photosynthesis, promoted root–shoot growth, and activated biosynthesis of medicinal compounds.

## 1. Introduction

Drought-induced water scarcity presents a significant and escalating global challenge, leading to profound disruptions in agricultural systems, substantial reductions in crop yields, and, in severe cases, widespread crop failure [1]. Climate change is driving increases in the frequency and intensity of drought events worldwide, posing a growing threat to sustainable agriculture [2]. Driven by global warming, the areas of arid and semi-arid regions in China have been expanding, now covering over 50% of the national land area, with these regions predominantly located in major agricultural zones such as north China and the northwest [3]. This expansion of arid regions not only threatens food security but also impacts the cultivation of medicinal plants, which are an essential part of traditional Chinese medicine. Under drought conditions, declining soil water potential restricts water uptake by roots, resulting in cellular dehydration, loss of turgor, reactive oxygen species (ROS) burst, membrane lipid peroxidation, electrolyte leakage, and ultimately, loss of membrane integrity [4]. Moreover, drought stress disrupts chloroplast ultrastructure, suppresses chlorophyll biosynthesis, and impairs photosynthetic function [5], collectively leading to growth inhibition, yield reduction, and even crop mortality [6]. Therefore, enhancing crop drought tolerance and elucidating the underlying mechanisms are imperative for safeguarding agricultural productivity.

*Prunella vulgaris* L., a renowned perennial plant used for both medicinal and culinary purposes, is named “Xia Ku Cao” in Chinese for its characteristic post-summer withering [7,8]. In traditional Chinese medicine (TCM), its dried fruit spicas are utilized for clearing heat, detoxifying, reducing swelling, and dissipating nodules [9]. Although the entire plant contains bioactive compounds, the fruit spikes exhibit the highest concentration of phenolic acids, flavonoids, and triterpenoids, justifying their use as the standardized medicinal part [10]. Among these, the highly abundant rosmarinic acid has been established as a key quality marker [11]. The pharmacological activities of these constituents are broad, encompassing not only anti-inflammatory, antioxidant, and antibacterial effects but also antitumor properties mediated through the inhibition of proliferation, cell cycle arrest, and induction of apoptosis [12]. Furthermore, its antihyperglycemic and cardioprotective effects, demonstrated in modern research, collectively underpin the pharmacodynamic basis of *P. vulgaris* as a source of promising natural therapeutic agents [7]. In China, *P. vulgaris* is mainly cultivated in Henan, Zhejiang, and Anhui provinces. These regions are subject to frequent seasonal droughts, which substantially compromise plant growth and ultimately reduce productivity [13,14]. With increasing market demand and a gradual shift from wild harvesting to cultivation, mitigating drought-induced yield losses has become a major agronomic and research priority.

Abiotic stresses, notably drought, severely constrain plant performance globally. To mitigate such impacts, exogenous plant growth regulators have become a key tool for enhancing stress resilience. Brassinosteroids (BRs), polyhydroxylated steroids, are critically involved in regulating plant growth, development, and stress responses [15]. Among them, 2,4-epibrassinolide (EBR), a synthetic BR with high bioactivity, is widely used in stress studies [16]. Research has shown that EBR application significantly increased biomass accumulation, chlorophyll content, and photosynthetic parameters in *Coriandrum sativum* seedlings [17]. Furthermore, under drought stress induced by 20% (*w*/*v*) PEG-6000 in a growth chamber, the application of EBR significantly enhanced the activities of antioxidant enzymes, such as SOD and POD, and it also promoted the synthesis of soluble sugars, soluble proteins, and chlorophyll in *P. vulgaris* cultured in Petri dishes [18]. However, the regulatory effects of EBR on *P. vulgaris* under drought stress remain largely unexplored and warrant further investigation.

Photosynthesis is vital for plants, where the light reactions and dark reactions work closely together to synthesize organic compounds through the conversion of light energy into chemical energy [19]. The light reactions, relying on the synergy of photosystem (PS) II and PS I, produce ATP and NADPH, which provide energy and substances for the dark reactions, driving the entire photosynthetic process to complete energy conversion and organic compound synthesis [20]. Drought stress severely disrupts the photosynthetic apparatus, but evidence suggests that EBR can protect photosynthetic function under adverse conditions [21]. In drought-stressed *Capsicum annuum* plants, EBR alleviated photoinhibition via a coordinated enhancement of electron transport in PSII and functional reaction centers, coupled with suppressed non-photochemical quenching [22]. Exogenous EBR treatment of *Rhododendron delavayi* under drought stress resulted in significant improvements in PSII function, as indicated by elevated values of capture efficiency (Fv′/Fm′), actual photochemical efficiency (φPSII), photochemical quenching coefficient (qP), and electron transfer rate (ETR) [21]. However, the effects of EBR on the coordinated activities of both PSI and PSII in *P. vulgaris* under drought remain poorly understood.

As the principal structures for water and nutrient uptake, roots exhibit exceptional sensitivity to fluctuations in soil water content [23]. Extensive research has consistently demonstrated that intense drought conditions directly compromise the functional performance and architectural integrity of root systems in plants, a process that significantly weakens the root capacity for water and nutrient absorption, thereby threatening the overall survival of plants [24]. In contrast, exogenous 24-epibrassinolide (EBR) application has been reported to confer a protective effect against drought-induced suppression of root growth. In *Echinacea purpurea*, EBR application under drought stress significantly increased root dry matter accumulation [25]; in *Glycine max* (soybean), EBR could optimize root spatial structure and physiological functions by promoting the development of key root tissues such as the epidermis, endodermis, and cortex, ultimately significantly enhancing plant drought [26]. Yet, the effects of EBR on the root architecture of *P. vulgaris* under drought stress remain to be elucidated.

Secondary metabolites, particularly phenolics and flavonoids, are crucial for plant adaptation to environmental stress and also determine the medicinal quality of *P. vulgaris* [27]. Studies have confirmed that Brassinosteroids (BRs), including exogenous applications of EBRs, effectively promote the accumulation of phenolic and flavonoid secondary metabolites in stressed plants, which consequently enhances their tolerance to abiotic stresses [28,29]. Under drought conditions, BRs promoted the accumulation of total phenols, flavonoids, and anthocyanins in *Camelina genus* [28] and *Glycine max* [29]. However, the regulatory effect of EBR on the secondary metabolism of *P. vulgaris* under drought conditions and the molecular mechanisms involved need further study.

Though earlier research has validated the capacity of brassinosteroid hormones (BRs) to relieve multiple non-biological stress factors, including high temperature [30] and drought [28,29], the physiological and biochemical mechanisms underlying EBR-mediated responses to drought stress in *P. vulgaris* have yet to be extensively investigated. Specifically, it is unclear how EBR influences photosynthetic performance, growth development, and biosynthesis of pharmaceutically valuable secondary metabolites in this species under drought conditions. Crucially, whether 24-epibrassinolide (EBR) can synergistically enhance drought tolerance, yield, and medicinal quality of *Prunella vulgaris* remains unelucidated—a core issue that is indispensable for the optimized cultivation of this species. This study therefore postulated that exogenous EBR, leveraging the established protective function of brassinosteroids, would alleviate oxidative stress and bolster physiological resilience in *P. vulgaris* under drought. To test this hypothesis, this study aimed to elucidate the effects of EBR on (i) antioxidant enzyme activities and osmotic regulatory substances; (ii) photosynthesis and chlorophyll fluorescence parameters; (iii) growth traits and biomass allocation; and (iv) secondary metabolites and key gene expressions. These results seek to elucidate the underlying mechanisms through which EBR strengthens drought resilience in *P. vulgaris*, thereby offering theoretical support for optimizing its cultivation practices and ensuring both yield and quality in water-deficient environments.

## 2. Results

### 2.1. Antioxidant Enzyme Activities and Phenylalanine Ammonia-Lyase Activity

**Figure 1:**
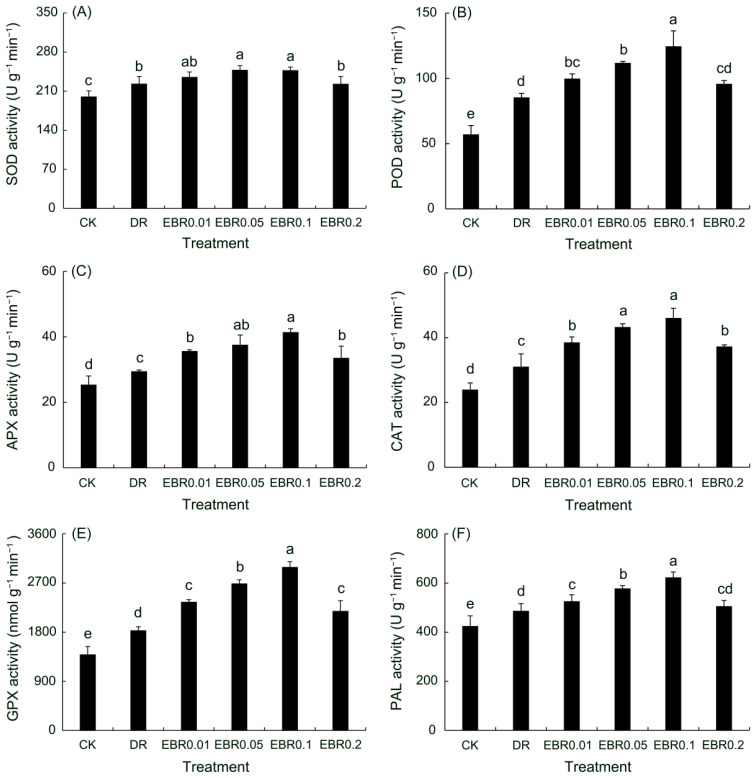
Effects of foliar-sprayed EBR at different concentrations on activities of SOD (**A**), POD (**B**), APX (**C**), CAT (**D**), GPX (**E**) and PAL (**F**) in drought-stressed *P. vulgaris* (Mean ± SD, n = 3, *p* < 0.05). Different lowercase letters above the bars denote significant differences.

Drought stress induced activation of the plant defense system, significantly elevating the activities of key antioxidant enzymes (SOD, POD, APX, CAT, GPX) and phenylalanine ammonia-lyase (PAL) to alleviate oxidative stress (Figure 1). EBR application further potentiated this defense response, synergistically enhancing the activities of these enzymes in a concentration-dependent manner to provide more effective protection against oxidative damage. The 0.1 μmol L^−1^ EBR treatment provided optimal enhancement of the enzymatic antioxidant system, achieving maximum functional capacity for ROS scavenging. Notably, the EBR0.2 treatment significantly mitigated the suppressive effect induced by drought stress, as evidenced by markedly higher activities of APX, CAT, and GPX relative to those in the drought-treated group.

### 2.2. Osmotic Regulators

**Figure 2:**
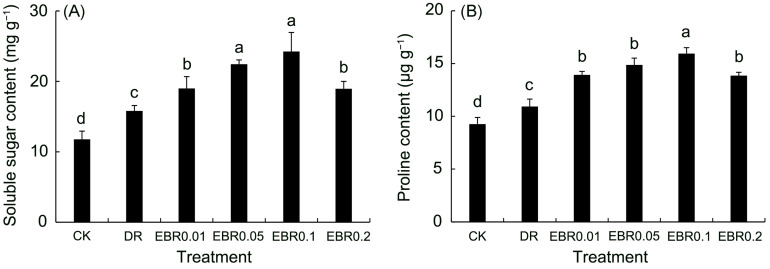
Effects of foliar-applied EBR at varying concentrations on the contents of soluble sugar (**A**) and proline (**B**) *P. vulgaris* under drought stress (Mean ± SD, n = 4, *p* < 0.05). Different lowercase letters above the bars denote significant differences.

In response to drought, Drought stress triggered a protective osmotic adjustment in *P. vulgaris* leaves (Figure 2), increasing soluble sugar by 34.31%, and proline by 17.88%. Foliar application of EBR further enhanced this adaptive response, with the EBR0.1 treatment being the most effective in promoting osmolyte accumulation, thereby better maintaining cellular water balance.

### 2.3. Leaf Relative Water Content and Soluble Protein

**Figure 3:**
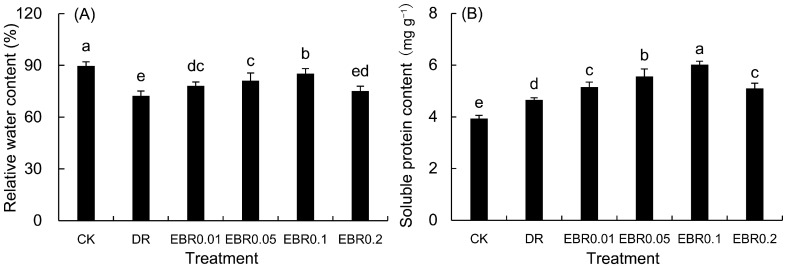
Effects of foliar-applied EBR at varying concentrations on leaf relative water content (**A**) and soluble protein content (**B**) of *P. vulgaris* under drought stress. Different lowercase letters indicate significant differences among treatments (*p* < 0.05). (Mean ± SD, n = 4 or 6, *p* < 0.05). Different lowercase letters above the bars denote significant differences.

Drought stress severely weakened the water retention function of *P. vulgaris* (Figure 3A), significantly reducing its leaf relative water content (RWC). However, exogenous EBR effectively counteracted this decline and improved leaf water status in a concentration-dependent manner, with the most pronounced mitigation observed at 0.1 μmol L^−1^. These results indicated that 2,4-epibrassinolide could effectively maintain the water balance in plants under drought stress.

Drought stress promoted the synthesis of soluble protein (Figure 3B), and EBR treatment further increased its content, reaching a peak under EBR0.1 treatment. The direct contribution of soluble proteins to cell osmotic potential is relatively limited, and their large accumulation under stress is often closely related to the activation of plant defense responses, protection of metabolic enzymes, and stability of cell structure.

### 2.4. Chlorophyll Content

**Figure 4:**
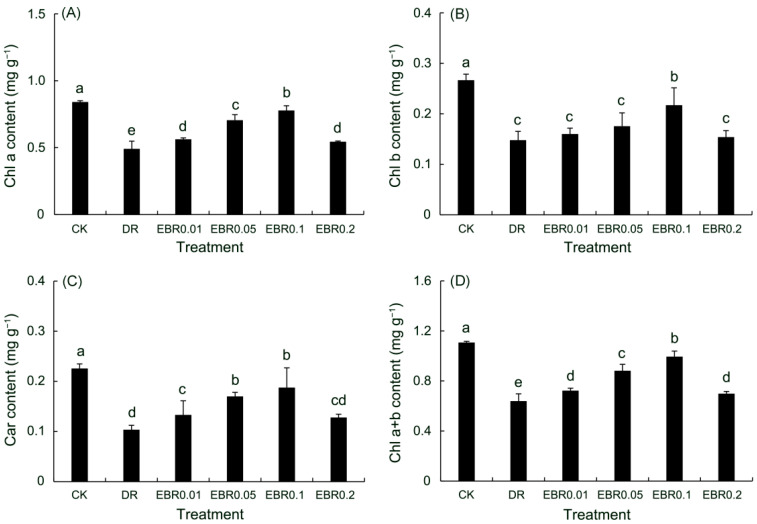
Effects of foliar application of different EBR concentrations on Chl a (**A**), Chl b (**B**), carotenoid (**C**) and total Chl a + b (**D**) contents in drought-stressed *P. vulgaris* (Mean ± SD, n = 4, *p* < 0.05). Different lowercase letters above the bars denote significant differences.

Drought stress significantly impaired the photosynthetic capacity of *P. vulgaris* leaves (Figure 4), resulting in reductions of 41.63% in chlorophyll a (Chl a), 44.52% in Chl b, 54.15% in carotenoids, and 42.33% in total chlorophyll (Chl a + b) in *P. vulgaris* leaves (*p* < 0.05). However, EBR pretreatment mitigated this damage. The EBR0.1 treatment demonstrated the best effect compared to drought control, optimally sustaining pigment levels to support photosynthetic performance during drought.

### 2.5. Oxidation Index

**Figure 5:**
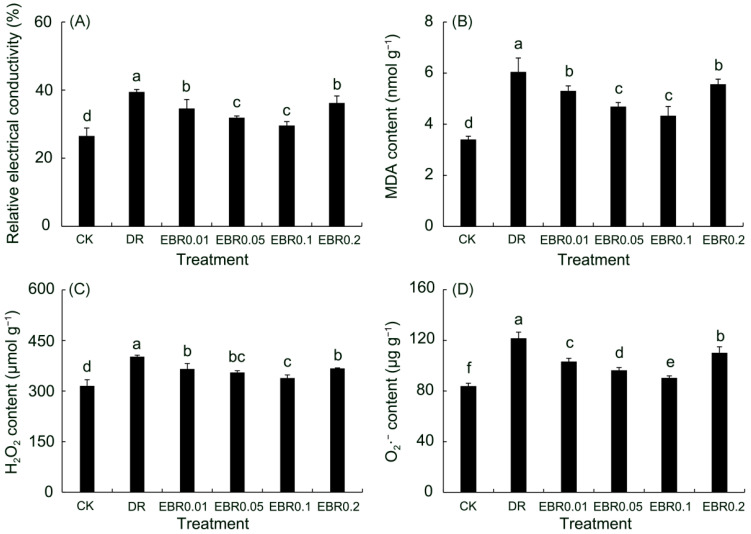
Effects of foliar spraying with different concentrations of EBR on REC (**A**), MDA (**B**) content, H_2_O_2_ (**C**) and O_2_^•−^ (**D**) content in *P. vulgaris* under drought stress (Mean ± SD, n = 4, *p* < 0.05). Different lowercase letters above the bars denote significant differences.

Drought stress induced severe oxidative damage, characterized by the accumulation of reactive oxygen species (O_2_^•−^, H_2_O_2_), a rise in MDA as a marker of membrane lipid peroxidation, and increased electrolyte leakage—a direct consequence of membrane integrity loss (Figure 5). EBR treatment effectively countered this damage by boosting the ROS-scavenging capacity, thus safeguarding membrane structure and function. The optimum response was achieved with EBR0.1 treatment, eliciting reductions of 25.01% in electrolyte leakage, 28.37% in MDA, 15.77% in H_2_O_2_, 25.73% in O_2_^•−^ relative to the DR group. Although EBR0.2 treatment was less effective than EBR0.1 treatment, it still kept all measured parameters above the drought control levels.

### 2.6. Gas Exchange Parameters

**Figure 6:**
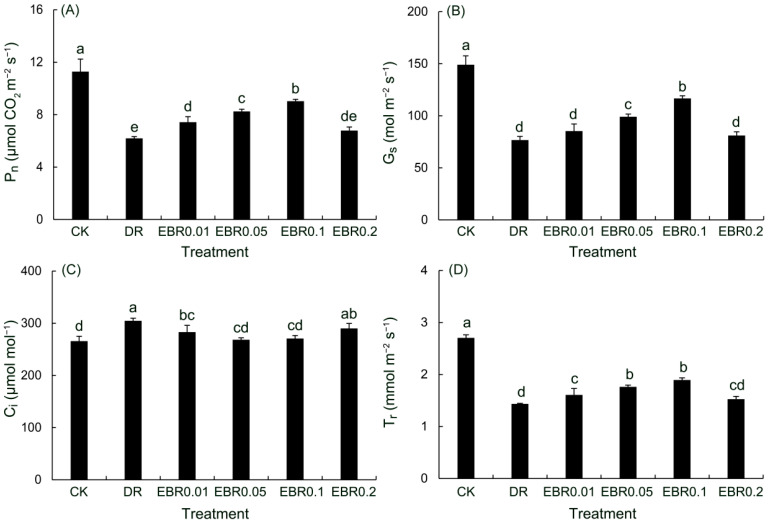
Effects of different concentrations of EBR on P_n_ (**A**), G_s_ (**B**), C_i_ (**C**) and T_r_ (**D**) of *P. vulgaris* under drought stress (Mean ± SD, n = 6, *p* < 0.05). Different lowercase letters above the bars denote significant differences.

Drought stress severely impaired photosynthetic gas exchange (Figure 6), reducing net photosynthetic rate (P_n_), stomatal conductance (G_s_) and transpiration rate (Tr), while increasing intercellular CO_2_ concentration (C_i_), indicating both stomatal and metabolic limitations. EBR application alleviated photosynthetic limitations by improving stomatal function (G_s_,T_r_) and enhancing the biochemical capacity for CO_2_ fixation (P_n_), thus restoring carbon assimilation. The most pronounced enhancement was achieved with EBR0.1 treatment, elevating P_n_, G_s_, and T_r_ by 45.88%, 52.17%, and 32.09%, respectively. Application of EBR resulted in a decrease in C_i_ across all concentrations tested, and the EBR0.05 and EBR0.1 treatment groups both exhibited substantial decreases, which were significantly greater than that of the drought-treated group, but the difference between these two treatment groups was not significant.

### 2.7. Rapid Chlorophyll Fluorescence Parameters

**Figure 7:**
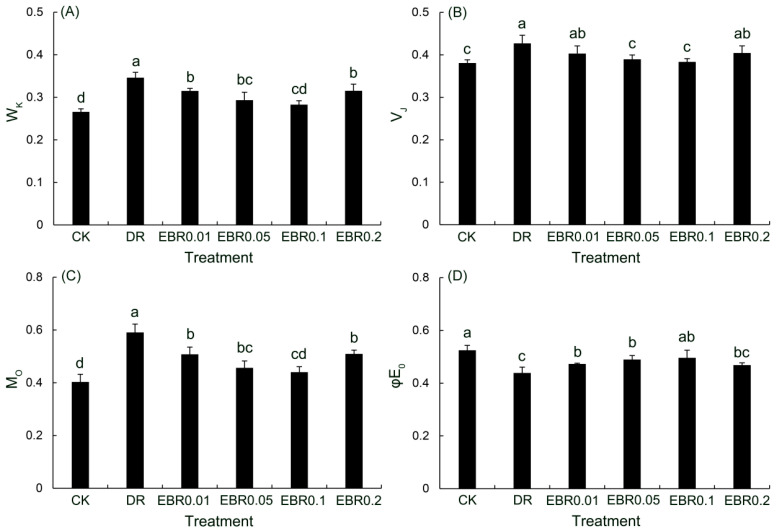
Effects of different concentrations of EBR on W_K_ (**A**), V_J_ (**B**), M_0_ (**C**) and ϕE_0_ (**D**) of *P. vulgaris* under drought stress (Mean ± SD, n = 10, *p* < 0.05). Different lowercase letters above the bars denote significant differences.

This study quantitatively analyzed the dynamic changes in the K and J phases of the fast chlorophyll fluorescence (OJIP) kinetics (Figure 7), using the normalized fluorescence at the K-step (W_K_) and the relative variable fluorescence at the J-step (V_J_) as core indicators. The results showed that drought stress significantly induced increases in W_K_, V_J_, and M_0_, while significantly decreasing the φE_0_ value, suggesting damage to the oxygen-evolving complex (OEC) and a blockage in the electron transport chain. EBR alleviated drought-induced photoinhibition by enhancing the electron transport efficiency of PSII and restoring the functionality of the oxygen-evolving complex (OEC), as evidenced by decreased W_K_, V_J_, and M_0_, and increased φE_0_. These improvements facilitated better electron flux from PSII to PSI, thereby maintaining photosynthetic coordination under drought. The most significant inhibition of these three parameters was observed under the 0.1 μmol L^−1^ EBR treatment. Similarly, φE_0_ exhibited a trend of initial increase followed by a decrease with increasing EBR concentration, also peaking at 0.1 μmol L^−1^.

**Figure 8:**
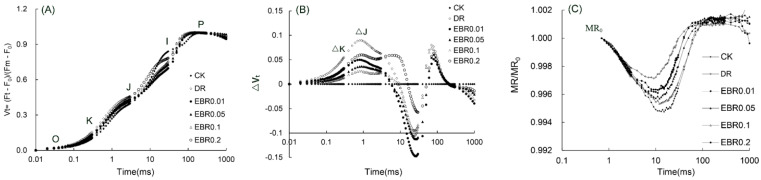
The Vt curve (**A**), △V_t_ curves (**B**), and 820-nm modulated reflection (**C**) in *P. vulgaris* under different EBR treatments. V_t_ = (F_t_ − F_0_)/(F_m_ − F_0_) denotes the relative variable fluorescence at time t; △V_t_ = V_t__(Se)_ − V_t__(CK)_; MR/MR_0_ represents the 820-nm modulated reflection signal, where MR is the reflectance at a given time and MR_0_ is the initial value under far-red light at 0.7 ms (Mean ± SD, n = 10, *p* < 0.05).

Drought stress significantly distorted the OJIP curve, evidenced by elevated K-step (t = 0.3 ms) and J-step (t = 2 ms) signals, and increased the minimum MR/MR_0_ value of the 820 nm modulated reflection, revealing comprehensive damage to the electron transport chain from PSII to PSI (Figure 8). EBR application, especially at 0.1 μmol L^−1^, most effectively reversed the changes in these key signals, proving EBR to be an effective strategy for maintaining the integrity of the photosynthetic electron transport chain.

### 2.8. Functions and Coordination of Photosystem II (PSII) and Photosystem I (PSI)

**Figure 9:**
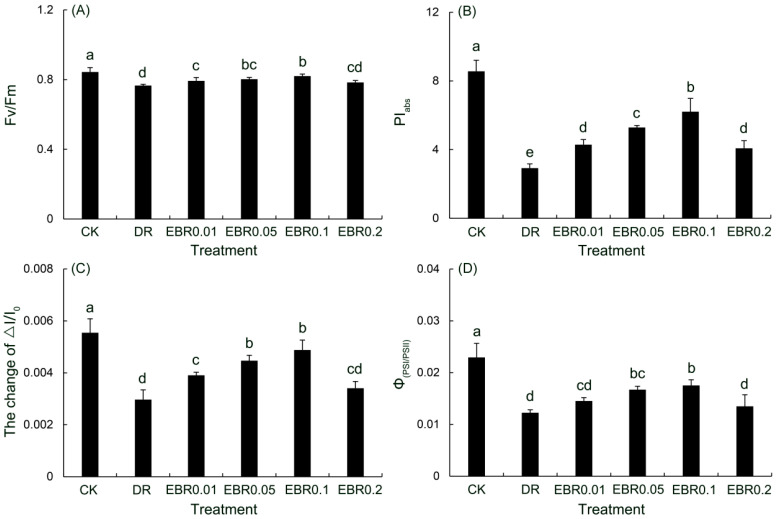
Effects of different concentrations of EBR on the functions and coordination of PSI and PSII in *P. vulgaris* under drought stress. Fv/Fm, (**A**); PI_abs_, (**B**); ∆I/I_0_, (**C**) and Φ_PSI/PSII_, (**D**) (Mean ± SD, n = 10, *p* < 0.05). Different lowercase letters above the bars denote significant differences.

Drought stress caused significant photoinhibition, reducing both the maximum quantum efficiency of PSII (F_v_/F_m_) and the overall photosynthetic performance index (PI_abs_) in *P. vulgaris* (Figure 9). Exogenous EBR restored these parameters, peaking at 0.1 μmol L^−1^. Exposure to drought also resulted in an inhibition of the maximum redox activity of PSI (ΔI/I_0_), concomitant with a decline in the coordination between PSII and PSI (Φ_PSI/PSII_). 0.01–0.1 μmol L^−1^ EBR elevated ΔI/I_0_ and Φ_PSI/PSII_, whereas 0.2 μmol L^−1^ EBR weakened this promotion, corroborating 0.1 μmol L^−1^ as the most effective concentration.

The rise in parameters representing energy fluxes per RC (ABS/RC, DI_0_/RC, TR_0_/RC, ET_0_/RC, DI_0_/CS_m_) under drought suggests a decline in energy use efficiency (Table 1). Conversely, the decline in parameters representing the performance parameters per cross-section (ABS/CS_m_, TR_0_/CS_m_, ET_0_/CS_m_, RC/CS_m_) indicates compromised damage to the closure of reaction centers and activity of the photosynthetic apparatus. EBR treatment effectively enhanced these parameters, with the 0.1 μmol L^−1^ treatment providing the best restoration of photosynthetic structure size and activity.

### 2.9. Growth Characteristics

Drought stress severely restricted root architectural development in *P. vulgaris*, impairing root elongation, surface area expansion, and branching capacity necessary for efficient water and nutrient acquisition compared to the control group (Table 2), as evidenced by reduced elongation, surface area, total volume, and the formation of root tips and lateral branches compared to CK treatment. EBR application mitigated drought-induced root suppression by promoting root elongation and branching, thereby enhancing soil exploration and water uptake capacity, with optimal effects at 0.10 μmol L^−1^.

For aboveground growth parameters of *P. vulgaris* (Table 3), drought stress markedly inhibited both vegetative and reproductive growth, as evidenced by reductions in stem length, branch number, spica traits, and overall biomass. Foliar EBR optimally restored drought-impaired shoot growth and yield parameters at 0.1 μmol L^−1^, following a bell-shaped response curve.

### 2.10. Secondary Metabolites

In response to drought-induced oxidative stress, *P. vulgaris* enhanced its phenolic biosynthesis for antioxidant protection (Table 4). This effect was further amplified by all EBR treatments, which promoted phenolic accumulation in a concentration-dependent manner, exhibiting an initial increase followed by a decrease. The maximum level was attained with 0.1 μmol L^−1^ EBR. Similarly, the biosynthesis of key medicinal compounds (caffeic acid, ferulic acid, rosmarinic acid, and hyperoside) was enhanced under drought stress, and further amplified by EBR treatment, with the most significant boost consistently observed at 0.1 μmol L^−1^ EBR treatment.

### 2.11. Gene Expression

**Figure 10:**
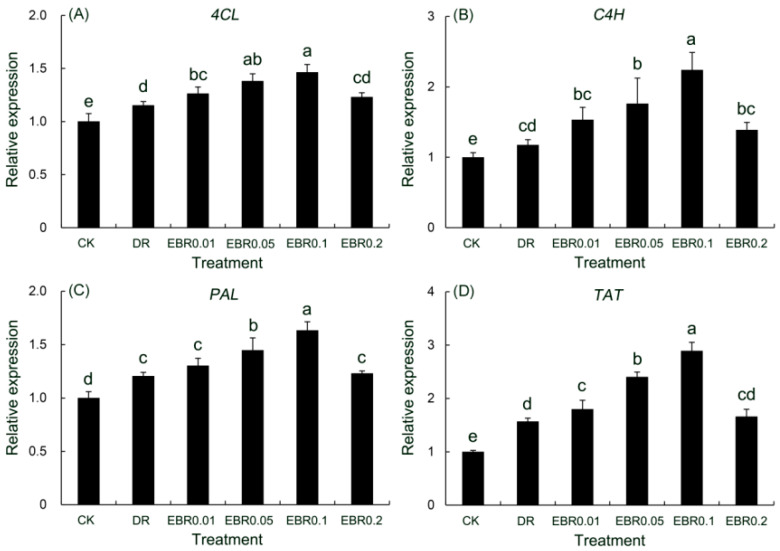
Relative expression levels of secondary metabolism-related genes in potted *P. vulgaris* after spraying EBR. (**A**) *4CL*: 4-coumaroyl CoA ligase; (**B**) *C4H*: coumarate 4-hydroxylase; (**C**) *PAL*: phenylalanine ammonia-lyase; (**D**) *TAT*: tyrosine aminotransferase. (Mean ± SD, n = 3, *p* < 0.05). Different lowercase letters are used to denote significant differences among treatments.

Drought stress transcriptionally activated key genes in the phenylpropanoid (*4CL*, *C4H*, *PAL*) and tyrosine-derived (*TAT*) pathways (Figure 10), with the *TAT* gene showing the most pronounced response. The promoting effect of EBR on the expression of key biosynthetic genes, which peaked at 0.1 μmol L^−1^, underpinned the concomitant peaking profile of medicinal compound production.

**Figure 11:**
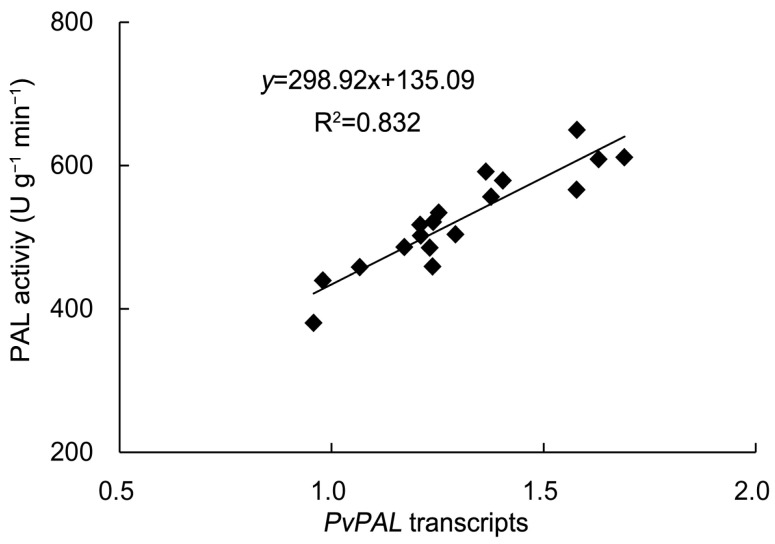
Correlation analysis between PAL enzyme activity and *PAL* gene transcription levels.

To elucidate the causal link between the EBR-induced upregulation of PAL transcripts and the enhancement of PAL enzyme activity, a correlation analysis was performed between PAL enzyme activity and *PvPAL* gene relative expression levels across the treatments. As shown in Figure 11, a significantly strong positive correlation was observed (R = 0.912, *p* < 0.01). This result statistically confirms that the transcriptional upregulation of the *PvPAL* gene is the direct cause of the increased PAL enzyme activity.

## 3. Discussion

### 3.1. Effects of EBR on Physiological Parameters

The activation of the antioxidant enzyme system observed in drought-stressed *P. vulgaris* seedlings might be attributed to elevated levels of ROS resulting from water deficit. Upon exposure to stress, plants typically activate endogenous antioxidant defense mechanisms to mitigate oxidative stress-induced cellular damage—a finding consistent with previous reports by Jang et al. [31]. Evidence from previous studies indicated that 24-epibrassinolide (EBR) application could augment the activities of CAT, SOD, and POD in drought-stressed *Echinacea purpurea* [25]. Furthermore, a significant promotive effect of EBR was observed on the activities of SOD, POD, CAT, and APX in *P. vulgaris* seedlings under hydroponic conditions with drought simulated by 20% PEG [18]. In line with these findings, foliar application of EBR similarly elevated the antioxidant enzyme activities in soil-cultured potted *P. vulgaris* seedlings subjected to drought stress in the present experiment.

Leaf relative water content (RWC) is a direct indicator of plant water status under drought stress [32]. The significant decline in RWC under drought stress confirmed the occurrence of cellular dehydration, which aligned with the observed reductions in photosynthetic efficiency and growth. Our results showed that EBR application effectively maintained higher RWC, which was consistent with the findings in maize that EBR improves drought tolerance by preserving water status [33]. This effect might be associated with EBR-induced optimization of root architecture, as enhanced root length, surface area, and root tip number can improve water uptake capacity under water-deficit conditions. Additionally, EBR-promoted accumulation of soluble sugars and proline might contribute to maintaining cellular turgor pressure, thereby reducing water loss and stabilizing RWC.

Plants enhance abiotic stress tolerance by maintaining cellular water and solute homeostasis [34]. Drought stress significantly increased the contents of soluble sugars in *P. vulgaris*, suggesting their potential role as osmoregulatory adaptations for maintaining cellular water balance [35]. Previous research demonstrated that drought stress significantly enriched differentially expressed genes in carbohydrate metabolism pathways [31], promoting the biosynthesis of osmolyte-related sugars such as raffinose and trehalose [36]. These factors are hypothesized to collectively account for the observed increase in osmolyte content in *P. vulgaris* under drought conditions. Similar responses were also reported in *Agropyron mongolicum* [37] under drought stress. This study demonstrated that exogenous application of EBR at various concentrations significantly enhanced the levels of soluble sugar and proline in *P. vulgaris* seedlings. This observed physiological regulation likely results from a coordinated interplay of several biochemical pathways. Prior research has shown that EBR markedly stimulates the key enzymes responsible for starch degradation, including α-amylase and β-amylase, which promote the breakdown of starch reserves in seedlings and provide ample substrates for soluble sugar synthesis [35]. Moreover, EBR promoted the biosynthetic activity of key sucrose-metabolizing enzymes such as sucrose phosphate synthase, consequently elevating the soluble sugar content in leaves [38]. Regarding proline metabolism, EBR transcriptionally activated key biosynthetic genes such as *CqproDH1, CqproDH2* and *CqOAT* [39], thereby facilitating net proline accumulation and significantly bolstering cellular osmotic adjustment capacity. The present study demonstrates that exogenous EBR application significantly enhances the accumulation of soluble sugars in the leaves of soil-cultured *P. vulgaris* under drought stress. This result aligns with the findings reported by Yao et al. [18], where EBR seed pretreatment under hydroponic conditions with PEG-induced drought similarly elevated the levels of these osmolytes. The consistency between this cross-experimental system (hydroponics vs. soil culture) and the treatment method (seed soaking vs. foliar spraying) strongly proves the stability of the physiological response of EBR to promote the accumulation of osmotic adjustment substances. These small-molecule solutes effectively reduced the water potential in the cells and ensured that the plants could still maintain a certain turgor pressure and physiological activity when the soil water potential was reduced, which was consistent with the results that the EBR treatment group had higher RWC.

In this study, drought stress promoted the accumulation of soluble proteins, most of which were functional proteins induced under drought, such as LEA proteins, antioxidant enzymes, and key enzymes in metabolic pathways [40]. These proteins are primarily associated with protective functions (stabilizing protein structures and protecting cell membranes), enhancing plant stress resistance by scavenging reactive oxygen species, maintaining membrane integrity, and reinforcing metabolic networks [41,42], rather than serving as the main osmoregulatory solute to reduce osmotic potential [43]. EBR-induced soluble protein accumulation may be attributed to its ability to inhibit proteolytic enzyme activity, thereby reducing protein degradation under drought stress [44]. Moreover, EBR treatment significantly increased the activity and gene expression of antioxidant enzymes such as SOD, POD, and CAT [42], as well as the expression of key enzyme genes in secondary generation pathways such as PvPAL, thereby enhancing the oxidative tolerance of plants. EBR enhances drought resistance through a dual mechanism: first, by maintaining cellular water status via osmotic adjustment; second, by upregulating the expression and function of defense proteins, thereby sustaining physiological stability under drought stress.

In *P. vulgaris*, 0.1 μM EBR was identified as the optimal concentration for elevating antioxidant enzyme activities and osmolyte content, whereas a reduced effect was observed at 0.2 μM EBR, suggesting a non-linear, dose-dependent relationship (Figure 1). This pattern contrasts with the linear dose–response reported in echinacea [25], a phenomenon potentially arising from the narrower range of concentrations tested in the latter study, which might have been insufficient to define the complete efficacy window of EBR.

### 3.2. Effects of EBR on Photosynthetic and Chlorophyll Fluorescence Parameters

Research has indicated that drought stress triggers substantial content of reactive oxygen species (ROS) in plants, thereby inducing membrane lipid peroxidation and ultimately compromising chloroplast membrane stability, which represses the expression of major chlorophyll synthesis genes, including *CHLI,* and stimulates chlorophyll breakdown genes like *Chlase*, finally leading to lowered chlorophyll levels [5,45]. Likewise, drought repressed the expression of carotenoid biosynthetic genes such as *DcPSY1* and *DcLCYB*, resulting in decreased carotenoid content [46]. These mechanisms accounted for the significant inhibition of photosynthetic pigments in *P. vulgaris* under drought, a phenomenon also observed in chickpea [47]. The application of EBR, however, effectively mitigated these detrimental effects. EBR was also shown to upregulate key genes involved in chlorophyll biosynthesis, such as *HEMC*, *HEMD*, and *HEME1* [48], and carotenoid synthesis genes, including *BoaCRTISO* and *BoaPSY2* [49], fostering the accumulation of both chlorophylls and carotenoids. Consequently, a notable increase in the content of total chlorophyll and carotenoids was observed in *P. vulgaris* leaves following EBR application under drought conditions, echoing reports of its positive effect on these photosynthetic pigments in *Carapa guianensis* under comparable stress [50], and paralleling the pigment accumulation observed in Petri-grown *P. vulgaris* seedlings treated with EBR during PEG-simulated drought [18].

Gas exchange analysis revealed that drought treatment significantly inhibited P_n_ and G_s_ in *P. vulgaris*, yet triggered a rise in C_i_. A similar trend of change was also observed in maize plants subjected to drought stress [51], indicating that non-stomatal limitation became the primary factor contributing to the reduction in P_n_ of *P. vulgaris*. Drought stress triggered substantial accumulation of ROS, which subsequently impaired the functionality of the oxygen-evolving complex on the donor side of photosystem II (PSII) and the associated electron transport chain in *P. vulgaris*. This impairment ultimately resulted in a decline in the activity of reaction centers (RC/CS_m_) and a reduction in electron transport efficiency (φE_0_). The significant decreases in Fv/Fm and PI_abs_ provided further evidence for the damage inflicted upon the photosynthetic apparatus. The experimental results indicated that EBR application effectively potentiated the activities of antioxidant enzymes in *P. vulgaris* subjected to drought conditions, effectively scavenged excess ROS, thereby alleviating oxidative damage and better preserving chlorophyll content. More importantly, EBR effectively increased RC/CS_m_ and φE_0_, and improved PSII performance under drought conditions. The increase in the indicator values of Fv/Fm and PI_abs_ clearly demonstrated a notable improvement in the operational efficiency of the photosynthetic apparatus, with this improvement further manifested by a marked rise in P_n_. These findings aligned with prior studies where brassinosteroid application improved photosynthetic capacity in drought-stressed *Rhododendron delavayi* [21] and *Lycopersicon esculentum* [52].

In *P. vulgaris* exposed to drought, W_K_ was significantly elevated relative to the control, indicating impaired oxygen-evolving complex (OEC) activity. M_0_ rose substantially, indicated an accelerated Q_A_ reduction rate, and hindered the electron transfer chain. An increase in M_0_ indicated a decrease in activity in the reaction, and the ability of Q_A_ to transfer electrons downwards was inhibited. The concomitant increase in V_J_ and decrease in φE_0_ confirmed that drought exerted an inhibitory effect on the electron transport ability at the acceptor side of PSII [53]. Consequently, the severely restricted electron transport resulted in electron leakage that reacted with O_2_, triggering a burst of ROS [54]. This oxidative assault compromised membrane integrity and increased electrolyte leakage, as evidenced by the significant increases in ROS content, MDA and REC in drought-stressed leaves. Application of 0.1 µM EBR markedly lowered W_K_, V_J_ and M_0_, while φE_0_ was significantly enhanced; ROS accumulation and electrolyte leakage were simultaneously decreased. These results showed that EBR effectively reduced electron leakage and ROS production, thereby preserving membrane stability under drought.

Drought stress markedly suppressed light harvesting, trapping and electron transport of PSII, resulting in photochemical impairment. Compared with control, drought decreased ABS/CS_m_, TR_0_/CS_m_ and ET_0_/CS_m_, indicating a severe restriction in photon absorption, trapping and electron transport capacity. Concomitantly, RC/CS_m_ also declined, compelling the remaining active reaction centers to enhance their efficiency to compensate for the overall loss, as reflected by significant increases in ABS/RC, TR_0_/RC and DI_0_/RC. However, ET_0_/RC and φE_0_ still dropped, whereas heat-dissipation indices DI_0_/CSm and φD_0_ rose greatly. These results suggested that drought-induced PSII dysfunction originated from simultaneous inhibition at both donor and acceptor sides, forcing the antenna to dissipate excessive excitation energy as heat in order to protect the electron transport chain from photoinhibitory damage [55]. Foliar pretreatment with 0.1 µM EBR significantly reversed the drought-induced alterations. EBR elevated RC/CS_m_, ET_0_/RC and φE_0_, decreased DI_0_/RC. In parallel, ABS/CS_m_, TR_0_/CS_m_ and ET_0_/CS_m_ at the cross-sectional level were increased. These parameter variations demonstrated that EBR treatment notably lowered the energy charge of each reaction center, which in turn minimized photoinhibition. Notably, in the EBR treatment, DI_0_/CSₘ and φD_0_ decreased, while ET_0_/CSₘ and RC/CSₘ increased significantly (Table 2), indicating that EBR application effectively minimized the dissipation of excess excitation energy, thereby facilitating the electron transport process and enhancing the photochemical activity of PSII [19,56].

Drought stress markedly reduced Fv/Fm and PI_abs_ in *P. vulgaris*. The synchronous decline of these two parameters indicated severe photoinhibitory damage and a significant deterioration in photosynthetic efficiency under drought stress. Exogenous 0.1 µM EBR greatly increased F_v_/F_m_ and PI_abs_, demonstrating that EBR effectively alleviated drought-induced PS II photoinhibition and substantially improved its photosynthetic efficiency. MR assays at 820 nm demonstrated that drought stress markedly suppressed ΔI/I_0_, concomitant with an elevation in MR/MR_0_ and a concomitant decline in the Φ_PSI/PSII_. These results collectively revealed that drought severely impaired PS I function and disrupted the coordination of electron transport between PS II and PS I. EBR treatment increased ΔI/I_0_ and Φ_PSⅠ/PSⅡ_, these results indicated that EBR application effectively optimized PSI performance while promoting intersystem electron transport between PSII and PSI, and effectively elevated the coordination between the two photosystems, thereby ameliorating photosynthetic performance under drought stress. Similarly, *Capsicum annuum* [21,22] and *R. delavayi* [21], drought stress reduced parameters such as F_v_/F_m_ and PI_abs_, and the application of EBR significantly improved these chlorophyll fluorescence parameters.

While previous studies on drought stress have primarily focused on PSII-related parameters such as Fv/Fm, PI_abs_ [21,22], the function of PSI and its coordination with PSII has remained largely unexplored. This study confirmed that under drought stress, 24-Epibrassinolide (EBR) treatment not only significantly improved the function of Photosystem II (PSII) in *P. vulgaris* but also effectively enhanced Photosystem Iactivity (ΔI/I_0_) and the coordination between PSII and PSI (Φ_PSI/PSII_). Strong support for this finding comes from Fang et al. [57]: brassinosteroids (BRs) mediate the accumulation of apoplastic hydrogen peroxide (H_2_O_2_) through the BZR1-RBOH1 pathway, thereby inducing cyclic electron flow (CEF) in PSI, maintaining the repair cycle of the PSII D1 protein, and ultimately enhancing the photoprotection of both PSI and PSII. In addition, the increase of Φ_PSI/PSII_ effectively coordinated the excitation energy distribution between the two photosystems, optimized the operation of the photosynthetic electron transfer chain, and prevented excessive excitation of PSII, thereby significantly reducing photoinhibition damage. This finding confirms that EBR confers systemic protection to the photosynthetic apparatus, extending beyond PSII. By safeguarding PSI function and promoting intersystem synergy, EBR maintains the efficiency and stability of the entire photosynthetic electron transport chain under stress, thereby improving photosynthetic performance. This provides crucial new evidence for a deeper understanding of the intrinsic mechanisms by which EBR enhances plant drought tolerance.

### 3.3. Effects of EBR on the Growth of P. vulgaris

The evidence obtained in this study revealed a significant inhibitory effect of drought stress on the growth and development of *P. vulgaris*—a wetland-adapted medicinal species naturally found in moist habitats such as riparian zones and valleys [27], which suggested an inherently low degree of drought tolerance [58]. Owing to its intrinsic susceptibility, prolonged drought in this study inflicted cumulative damage on the root system of *P. vulgaris*, ultimately limiting aerial growth and reproductive development. Furthermore, impaired photosynthetic efficiency, limited nutrient uptake, and substantial oxidative damage collectively constrained carbon assimilation and its allocation to sink organs, ultimately leading to the observed reduction in biomass under drought conditions. The results of this investigation not only validated the generality of the growth-inhibitory effect of drought stress on *Glycyrrhiza* species as reported by Chang et al. [59], but were also consistent with the documented negative impact of drought on yield formation in *P. vulgaris* observed by Guo et al. [58].

The application of EBR exogenously effectively mitigated the adverse effects of drought on *P. vulgaris*. The ameliorative effects of EBR were likely mediated through the activation of brassinosteroid signaling pathways, modulating genes involved in cell division and elongation [60], which then promoted the elongation of primary roots and the initiation of lateral root primordia [61], thereby boosting its efficiency in taking up water and nutrients. Additionally, EBR notably improved the photosynthetic capacity of *P. vulgaris* under drought stress, potentially through increasing stomatal conductance and the activity of antioxidant enzymes, which alleviated photoinhibition and oxidative damage, consequently enhancing carbon assimilation efficiency and promoting the transport of photoassimilates to spicas. Consistent with these observations, analogous positive effects of EBR on photosynthetic efficiency and biomass accumulation under drought were also reported in maize [51].

The growth promotion by EBR observed in our soil-cultured *P. vulgaris*, though consistent in direction, showed quantitative distinctions from reports in PEG-hydroponic systems with seed priming [18]. We propose that these differences stem from the integrated effects of cultivation method and EBR application strategy. The gradual stress development in soil contrasts with the rapid PEG-imposed dehydration, likely altering the temporal pattern of EBR-mediated responses. Moreover, foliar application to mature plants targets a fully developed photosynthetic apparatus and hormonal network, potentially leading to a different efficacy profile compared to priming at the seed stage, which primarily prepares the plant for future stress. Thus, the experimental context—specifically, a soil-based system with foliar EBR—elucidates a distinct manifestation of EBR-induced growth enhancement under drought.

As a wetland medicinal plant with low inherent drought tolerance [27], *P. vulgaris* experiences significant inhibition of root and shoot growth under drought stress. Our study revealed that EBR (optimally at 0.1 µmol L^−1^) significantly alleviated these adverse impacts, comprehensively promoting both root morphology and aboveground development. In contrast to many studies that primarily focus on aboveground growth [51,58], our detailed quantification of root morphology directly highlights the key role of EBR in enhancing drought resistance from the basic sources of water and nutrient absorption. In addition, the observed positive regulation of osmotic adjustment substances, antioxidant system and photosynthetic efficiency together provide a physiological basis for the growth promotion of EBR in *P. vulgaris*.

### 3.4. The Effects of EBR on Secondary Metabolites and Related Gene Expression

*PvPAL* initiates the phenylpropanoid pathway by deaminating phenylalanine to produce cinnamic acid, which serves as a common precursor for phenolics and flavonoids. Subsequently, *PvC4H* and *Pv4CL* sequentially catalyze the hydroxylation of cinnamic acid and the synthesis of coumaroyl-CoA, connecting downstream branches [62]. Meanwhile, *PvTAT* indirectly boosts phenolics and antioxidants by regulating tyrosine metabolism to scavenge ROS and alleviate oxidative damage, thereby enhancing drought tolerance.

According to Wang et al. [63], *PvTAT* enhances the biosynthesis of phenolics and antioxidants indirectly by modulating the tyrosine metabolism pathway, thereby aiding in ROS clearance and mitigating oxidative damage to improve drought resistance. Furthermore, studies have confirmed that drought stress can activate the expression of genes associated with secondary metabolism, promoting the synthesis of compounds such as phenolics, flavonoids, and terpenoids, which contribute to ROS scavenging and enhance plant stress resistance [28,64]. Supporting these findings, our experimental results showed that the contents of several secondary metabolites—total phenolics, caffeic acid, ferulic acid, rosmarinic acid, and hyperoside—were significantly elevated in *P. vulgaris* under drought treatment. These metabolic changes were strongly supported by the significantly upregulated expression of *PvPAL*, *PvC4H*, *Pv4CL*, and *PvTAT* genes, which was consistent with the findings in chrysanthemum that drought upregulates key genes such as *PAL*, *CHI*, and *F3H* to promote the accumulation of phenolics and flavonoids like chlorogenic acid, luteolin, quercetin, and rutin [65]. Furthermore, investigations in mulberry also corroborated that drought stress significantly upregulated the transcription of key genes within the phenylpropanoid pathway, including *MaPAL*, *Ma4CL*, and *MaCHS*, leading to the accumulation of secondary metabolites such as quercetin derivatives and kaempferol [66]. Exogenous application of EBR further amplified the transcriptional upregulation of these target genes, with the 0.1 μmol L^−1^ EBR treatment eliciting the most pronounced enhancement in transcript abundance. The relative quantitative expression levels of these key genes exhibited a strong correlation with the fluctuations in medicinal component content within *P. vulgaris*, and the elevated PAL enzyme activity provided direct biochemical corroboration. The significant positive correlation and synchronous upregulation between *PvPAL* gene expression and PAL enzyme activity induced by EBR in our study align closely with observations on *AtPAL* 1/2 in *Arabidopsis*, where a strong positive correlation (R = 0.94, *p* < 0.01) between their transcripts and PAL activity, along with their stress-induced co-expression, drives flavonoid (e.g., kaempferol) accumulation [67]. This consensus indicates that the transcriptional regulation of *PAL*, as a key rate-limiting enzyme of the phenylpropanoid pathway, is a universal mechanism across species. Consequently, our results strongly demonstrate that the core mechanism for EBR-enhanced phenolic biosynthesis in *P. vulgaris* lies in the transcriptional activation of the *PvPAL* gene. This conclusion corroborated findings from watermelon studies, in which EBR upregulated the transcription of key phenylpropanoid biosynthetic genes, including *PAL* and *4CL*, leading to increased contents of total phenolics, flavonoids, and lignin [68,69]. EBR enhanced the biosynthesis of phenolic compounds by upregulating key phenylpropanoid pathway genes (*PvPAL*, *PvC4H*, *Pv4CL*, *PvTAT*), which not only contributed to ROS scavenging but also improved drought tolerance through enhanced antioxidant capacity and membrane stability. The work by Liang et al. [69] provided additional support for this notion, demonstrating that exogenous EBR significantly enhanced the enzymatic activities of key phenylpropanoid pathway enzymes, including *PAL*, *C4H*, and *4CL* in *Mongolian astragalus* seedlings under drought stress, thereby promoting flavonoid accumulation and enhancing their drought tolerance. In summary, EBR significantly enhanced the synthesis of secondary metabolites in *P. vulgaris* by upregulating the expression of multiple genes involved in the phenylpropanoid metabolic pathway, thereby improving its drought adaptability.

Although previous studies have confirmed that EBR promotes the accumulation of secondary metabolites in plants under drought stress [69], this study reveals a more specific regulatory mechanism in *P. vulgaris.* Similarly to the reported activation of secondary metabolic pathways by EBR in plants such as *M. astragalus* [69] and watermelon [68], our research demonstrated that EBR significantly increased the contents of total phenols, caffeic acid, ferulic acid, rosmarinic acid, and hyperoside in *P. vulgaris*, while upregulating key genes including *PAL*, *C4H*, and *4CL*. However, the uniqueness of this study lies in the discovery of particularly pronounced upregulation of the *TAT* gene by EBR, providing crucial molecular evidence for the substantial accumulation of phenolic compounds such as rosmarinic acid. This indicates that EBR not only generally enhances secondary metabolic pathways but also precisely regulates the metabolic response of *P. vulgaris* to drought stress through specific activation of *TAT*, thereby optimizing the synthesis of specific medicinal components. This discovery deepens our understanding of the mechanisms by which EBR regulates plant secondary metabolism and offers new perspectives on the unique responsiveness of *P. vulgaris* to EBR.

Notably, the accumulation of secondary metabolites in the control group of this study was lower than the findings of Chang et al. [19,70], which is likely to reflect the important effect of environmental differences on metabolic pathways. Numerous studies have verified that the accumulation of secondary metabolites is jointly restricted by multiple factors, including light, temperature, soil moisture, fertility, and salinity, and the independent variation in any single factor can significantly change their content [71]. Consistent with the reported positive correlation between light intensity and secondary metabolite accumulation such as flavonoids and proanthocyanidins [72], *Centella asiatica* grown in full sunlight showed markedly higher production of saponins (asiaticoside, madecassoside), flavonoids, and chlorogenic acid than those in shaded environments [73], whereas low light caused a decline in flavonoid content in *Catharanthus roseus* leaves [74] and a marked decrease in total phenol content in *Aralia elata* [75]. The shading nets and rain shelters installed in this experiment significantly reduced light intensity, which might have inhibited the phenylpropanoid metabolic pathway, thus leading to a relatively low overall content of metabolites. In addition, harvest time was also a crucial factor influencing the accumulation of secondary metabolites. Studies indicated that the content of active ingredients in *P. vulgaris* decreased significantly with delayed harvesting [76], and the harvest time of this study was later than that of previous studies [19,70], which might have further lessened the accumulation of metabolites.

### 3.5. EBR Perception and Signaling in Transcriptional Regulation of Phenylpropanoid and Antioxidant Pathways

EBR, a brassinosteroid, is perceived by the plasma membrane receptor *BRI1*. This perception triggers the formation of an active heterodimer with the co-receptor BAK1, which launches the intracellular signaling cascade [77,78]. This signal is transduced through a phosphorylation cascade that inhibits the kinase BIN2, leading to the dephosphorylation and activation of key transcription factors such as *BZR1/BES1*, which then bind to BRRE or E-box elements in the promoters of target genes, thereby driving a transcriptional reprogramming that governs plant growth and stress responses [79,80].

Our study demonstrated that EBR treatment activated the phenylpropanoid pathway by significantly upregulating the expression of key genes, including *PvPAL*, *PvC4H*, *Pv4CL*, and *PvTAS*. This process is directly mediated by BZR/BES transcription factors, whose core mechanism involves the specific recognition and binding of BES1/BZR1 to E-box (CANNTG) or BRRE (CGTGT/CG) elements in the promoters of downstream target genes, thereby promoting their expression [68,81,82]. The conservation of this BR-mediated regulatory pathway across species is evidenced in watermelon, where exogenous BRs activate phenylpropanoid pathway genes through the identical mechanism [68]. Similarly, genes involved in the tyrosine metabolism pathway are also under the transcriptional control of BR signaling, which likewise influences the biosynthesis of phenolic compounds [63,83].

EBR significantly enhanced the antioxidant enzyme activities in *Prunella vulgaris*, which may be attributed to the transcriptional activation of related antioxidant genes. Studies have shown that brassinosteroids (BRs) can upregulate certain transcription factors that positively regulate the expression of antioxidant genes [84]. Furthermore, the BR signaling pathway can engage in crosstalk with other stress signals, such as ABA and SA, to coordinately regulate transcriptional responses to abiotic stress [85,86]. Hence, EBR sensing initiates a signaling cascade leading to the activation of specific transcription factors that upregulate antioxidant defense genes, thereby enhancing oxidative stress tolerance under drought conditions.

### 3.6. Novel Insights into EBR-Mediated Drought Resilience: Integrating Yield and Quality Enhancement

**Figure 12:**
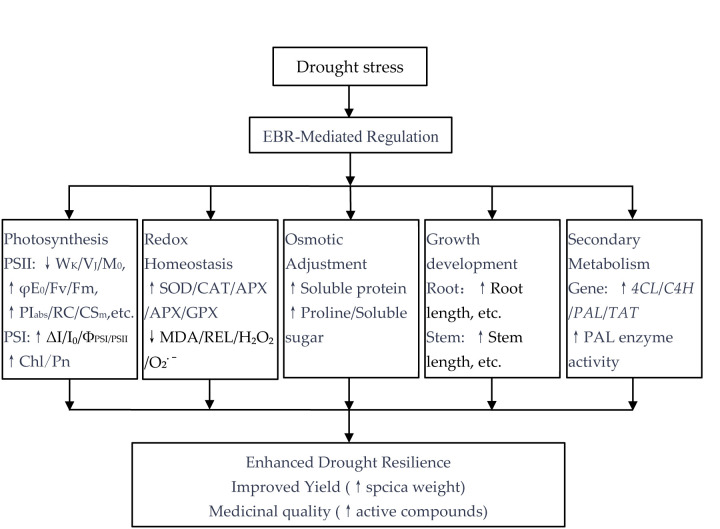
Schematic model of EBR-enhanced drought tolerance in *P. vulgaris*.

While the drought stress-mitigating effects of EBR have been reported in diverse crop species, this study provides novel mechanistic and applied insights specific to the medicinal plant *P. vulgaris* (Figure 12). First, our findings demonstrate that EBR functions beyond a mere stress survival strategy. By concurrently upregulating antioxidant defense systems, optimizing the coordination efficiency between photosystem I (PSI) and photosystem II (PSII) (Φ_PSI/PSII_), and specifically activating the phenylpropanoid-tyrosine pathway via key genes *PvPAL*, *PvC4H*, *Pv4CL*, and *PvTAT*, EBR uniquely converts a drought stress period into an opportunity for quality improvement. This coordinated regulatory response not only sustains biomass accumulation but also drives a significant increase in pharmaceutically active compounds, such as rosmarinic acid—a critical quality marker for *P. vulgaris*. Second, from an application-oriented perspective, we precisely identified 0.1 μmol L^−1^ as the optimal EBR concentration, which maximizes both spica yield and secondary metabolite content. This outcome offers a concrete, field-implementable agronomic practice for *P. vulgaris* cultivation in drought-prone regions, directly addressing the core challenge of maintaining medicinal quality under abiotic stress.

## 4. Materials and Methods

### 4.1. Plant Materials

Healthy seeds of *P. vulgaris*, uniform in size, plump, and free from pest damage, were selected and surface-sterilized. In October 2023, surface-sterilized seeds were uniformly sown into plastic nursery trays filled with a standard seedling substrate. The seeds were subsequently cultivated under routine irrigation in a controlled environment. In early April 2024, upon reaching an approximate height of 6 cm, the seedlings were transplanted into plastic pots measuring 24 cm in diameter and 26 cm in height. A growth substrate, prepared by mixing loam and peat in a 7:1 (*v*/*v*) ratio, was dispensed in equal volumes into each pot, with seven seedlings of *P. vulgaris* per pot. Commencing on April 15, the plants received Hoagland nutrient solution at five-day intervals. Each pot was irrigated with 1 L of the solution per application, for a total of three applications throughout the treatment period.

### 4.2. Experimental Design

The drought stress experiment was performed on *P. vulgaris* during the flowering stage in May 2024. Potted seedlings with uniform growth were selected as experimental materials, with 10 pots per treatment. Six treatments were set up in the experiment: (1) control + 0 μmol L^−1^ EBR solution (CK), (2) Drought group + 0 μmol L^−1^ EBR solution (DR), (3) Drought group + 0.01 μmol L^−1^ EBR solution (EBR0.01), (4) Drought group + 0.05 μmol L^−1^ EBR solution (EBR0.05), (5) Drought group + 0.1 μmol L^−1^ EBR solution (EBR0.1), and (6) Drought group + 0.2 μmol L^−1^ EBR solution (EBR0.2). Prior to experimentation, all potted seedlings were transferred to a rain-out shelter equipped with shade nets for a one-week acclimatization period. Following acclimatization, the plants were subjected to three foliar spray applications, administered at two-day intervals, immediately preceding the induction of drought stress. All pots were randomly rearranged every three days to ensure uniform light, temperature, and humidity conditions within the rain-out shelter. At 18:00 daily, each pot received 50 mL of its assigned EBR solution, all containing 0.1% Tween-80 for improved adhesion. To ensure uniformity in foliar treatments, equal volumes of deionized water with the same surfactant were applied to both CK and DR groups. Soil moisture levels were subsequently managed gravimetrically: CK was kept at 75% ± 5% field capacity (FC), whereas DR and EBR treatments were maintained under moderate water deficit conditions at 60% ± 5% FC. Daily water loss was replenished at 18:00 h by gravimetric adjustment, the experiments were conducted in pots under controlled conditions, and the gravimetric method with 5% tolerance was employed to ensure experimental consistency. All pots were randomly rearranged every three days to ensure uniform light, temperature, and humidity conditions within the rain-out shelter. After 20 days of drought treatment, various physiological indices were measured. Fruit spicas of *P. vulgaris,* harvested in late June, were first air-dried in the shade at room temperature for 12–48 h. Subsequently, they were oven-dried at a constant temperature of 50 °C until a constant mass was achieved and passed through a 60-mesh sieve to obtain a fraction for subsequent analysis of bioactive compounds.

### 4.3. Determination of Antioxidant Enzyme Activity

SOD activity was assayed according to the photochemical method previously described by Alam et al. [87]. Fresh leaves of *P. vulgaris* were harvested, snap-frozen in liquid nitrogen (N_2_), and ground to a fine powder. The resulting powder was extracted with ice-cold 0.05 mM phosphate-buffered saline (PBS, pH 7.8). Following centrifugation at 3000× *g* for 15 min at 4 °C, the supernatant was collected for SOD activity determination. For the assay, the enzyme extract was mixed with a reaction solution containing 0.05 M PBS, 130 mM methionine, 100 μM EDTA-Na_2_, 750 μM NBT, and 20 μM riboflavin. The reaction was initiated by exposure to light (4000 lx) for 20 min, after which absorbance was spectrophotometrically determined at 560 nm. Leaf samples for SOD analysis were collected from three potted *P. vulgaris* groups (n = 3); five leaves of the same nodal position and developmental stage were pooled per pot to constitute one biological replicate.

The activities of peroxidase (POD), ascorbate peroxidase (APX), and catalase (CAT) were quantified using the established methodologies [50]. For POD activity determination, the enzyme extract was incubated in 0.05 mol·L^−1^ phosphate buffer (pH 6.0) containing guaiacol and 30% H_2_O_2_. Absorbance was immediately measured at 470 nm at room temperature following the addition of the substrate. For APX activity, the enzyme solution was thoroughly mixed with 50 mmol·L^−1^ phosphate buffer (pH 7.0), 15 mmol/L AsA, and 0.3 mmol·L^−1^ H_2_O_2_. A blank was prepared by replacing the enzyme solution with phosphate buffer, and the absorbance change was measured at 290 nm over 3 min. For CAT activity, the enzyme extract was mixed with 0.05 mol·L^−1^ phosphate buffer (pH 7.8) and hydrogen peroxide solution. After reacting for 4 min, the reaction was terminated with 8% sulfuric acid, and the absorbance was measured using a quartz cuvette. Leaf samples for POD, APX and CAT analysis were collected from three potted *P. vulgaris* groups (n = 3); five leaves of the same nodal position and developmental stage were pooled per pot to constitute one biological replicate.

Glutathione peroxidase (GPX) activity was determined using a commercially available assay kit (Suzhou Grace Biotechnology Co., Ltd., Suzhou, China). Fresh leaves were weighed and immediately snap-frozen in liquid nitrogen before being pulverized. The resulting powder was homogenized in a pre-chilled extraction buffer. Following centrifugation of the homogenate at 12,000× *g* for 10 min at 4 °C, the supernatant was collected. Enzyme activity was then quantified by measuring the absorbance of the yellow product generated from the reaction of glutathione with DTNB at 412 nm. Leaf samples for GPX analysis were collected from three potted *P. vulgaris* groups (n = 3); five leaves of the same nodal position and developmental stage were pooled per pot to constitute one biological replicate.

Phenylalanine ammonia-lyase (PAL) activity was assayed according to a modified protocol [88]. Fresh leaves were homogenized in an ice-cold borate buffer (7 mmol·L^−1^ mercaptoethanol) and centrifuged at 12,000× *g* for 15 min at 4 °C. The resulting supernatant was collected for enzymatic assays. For PAL activity determination, the supernatant was combined with 0.1 mol·L^−1^ borate buffer (pH 8.8) and 0.02 mol·L^−1^ L-phenylalanine. Enzymatic activity was calculated based on the change in absorbance at 290 nm after a 30-min incubation at 30 °C. Leaf samples for PAL analysis were collected from three potted *P. vulgaris* groups (n = 3); five leaves of the same nodal position and developmental stage were pooled per pot to constitute one biological replicate.

### 4.4. Determination of Osmotic Adjustment Substances Content

Soluble sugar content was quantified in *P. vulgaris* leaves using the anthrone sulfuric acid method [89]. Fresh leaves were washed, homogenized, and extracted in boiling water. After cooling, anthrone-ethyl acetate reagent and concentrated sulfuric acid were added, and the mixture was vortexed, sealed, and reheated. Absorbance was then measured at 630 nm against a blank. Leaf samples for soluble sugar analysis were collected from four potted *P. vulgaris* groups (n = 4); A minimum of six leaves of the same nodal position and developmental stage were pooled per pot to constitute one biological replicate.

The determination of proline was conducted using the acid-ninhydrin method of Rezayian et al. [90]. A weighed fresh leaf sample was extracted with 3% sulfosalicylic acid via a 10-min boiling water bath. The supernatant obtained after centrifugation was incubated with the acid-ninhydrin reagent in a boiling water bath for 30 min. The resultant red chromophore was subsequently extracted into toluene, and its absorbance was determined spectrophotometrically at 595 nm. Sugar. Leaf samples for proline analysis were collected from four potted *P. vulgaris* groups (n = 4); A minimum of six leaves of the same nodal position and developmental stage were pooled per pot to constitute one biological replicate.

### 4.5. Determination of Leaf Relative Water Content (RWC) and Soluble Protein

Leaf relative water content (RWC) was measured following the method of Ghobadi et al. [91] with slight modifications. Specifically, the fresh weight (Wf) of the sample was recorded. The sample was then saturated in distilled water for 24 h, and the turgid weight (Wt) was measured. After that, the sample was dried at 80 °C to constant weight to determine the dry weight (Wd). RWC was calculated as (Wf − Wd)/(Wt − Wd) × 100%. Each treatment was conducted with six biological replicates.

Soluble protein concentration was determined according to the method described by Zhang et al. [92]. Leaf tissue (0.20 g) was homogenized in 8 mL of 1% (*w*/*v*) PVP solution, and centrifuged at 1000 × rpm for 10 min at 4 °C. The resulting supernatant was then mixed with Coomassie Brilliant Blue G-250 reagent. Absorbance, measured at 595 nm after a 2-min incubation at room temperature, was used to calculate protein concentration. Leaf samples for soluble protein analysis were collected from four potted *P. vulgaris* groups (n = 4); A minimum of six leaves of the same nodal position and developmental stage were pooled per pot to constitute one biological replicate.

### 4.6. Determination of Photosynthetic Pigment Contents

The concentrations of photosynthetic pigments, including chlorophyll a, chlorophyll b, total chlorophylls, and carotenoids, were determined via a 95% ethanol extraction protocol [18]. Briefly, fresh leaves of *P. vulgaris* were subjected to homogenization and immersion in 95% ethanol, followed by a 48-h extraction period in complete darkness. To quantify the respective pigment contents, the absorbance of the resulting extracts was measured spectrophotometrically at 470 nm, 649 nm, and 665 nm. Leaf samples for pigment analysis were collected from four potted *P. vulgaris* groups (n = 4); A minimum of six leaves of the same nodal position and developmental stage were pooled per pot to constitute one biological replicate.

### 4.7. Determination of Oxidative Stress Indicators

MDA content was quantified by the thiobarbituric acid (TBA) method of Wang et al. [93]. Following homogenization of 0.3 g fresh leaves in 5% trichloroacetic acid (TCA) on ice and centrifugation, the supernatant was reacted with 0.67% TBA via a 30-min incubation in a boiling water bath. The absorbance of the cooled and centrifuged supernatant was determined spectrophotometrically at 450 nm, 532 nm, and 600 nm to quantify MDA. Leaf samples for MDA analysis were collected from four potted *P. vulgaris* groups (n = 4); A minimum of six leaves of the same nodal position and developmental stage were pooled per pot to constitute one biological replicate.

The O_2_^•−^ content was determined using the hydroxylamine method [94]. Following the preparation of a leaf homogenate in 65 mM PBS (pH 7.8), filtration, and centrifugation, the supernatant was reacted with hydroxylamine hydrochloride at 25 °C for 20 min. Following the sequential addition of sulfanilic acid and α-naphthylamine, the mixture was incubated at 30 °C for 30 min to allow for color development. Absorbance was then measured colorimetrically at 530 nm. Leaf samples for O_2_^•−^ analysis were collected from four potted *P. vulgaris* groups (n = 4); A minimum of six leaves of the same nodal position and developmental stage were pooled per pot to constitute one biological replicate.

The determination of H_2_O_2_ content was based on the method of Zhang et al. [95]. Cryogenic grinding in liquid nitrogen and homogenization in ice-cold 0.1% TCA yielded a leaf extract. After centrifugation, the supernatant was incubated with phosphate buffer (pH 7.0) and KI in darkness for 1 h, and the absorbance was subsequently measured spectrophotometrically at 390 nm. Leaf samples for H_2_O_2_ analysis were collected from four potted *P. vulgaris* groups (n = 4); A minimum of six leaves of the same nodal position and developmental stage were pooled per pot to constitute one biological replicate.

Based on Ghobadi et al. [91], relative electrical conductivity (REC) was determined. Fresh leaf segments were submerged in a precisely determined volume of deionized water. After incubating at room temperature for 24 h with gentle agitation, the initial electrical conductivity (R1) of the bathing solution was recorded. The samples were then boiled for 30 min and cooled, and the final conductivity (R2) was measured. REC was calculated as (R1/R2) × 100%. Leaf samples for REC analysis were collected from four potted *P. vulgaris* groups (n = 4); A minimum of six leaves of the same nodal position and developmental stage were pooled per pot to constitute one biological replicate.

### 4.8. Measurement of Photosynthetic and Chlorophyll Fluorescence Parameters

Photosynthetic gas exchange parameters were determined between 08:00 and 11:00 on clear days using a TARGAS-1 portable photosynthesis system (PP Systems, Amesbury, MA, USA). The third and fourth fully expanded leaves were sampled per treatment. Instrumental parameters including airflow (250 cc min^−1^), photosynthetically active radiation (PAR, 1000 μmol m^−2^ s^−1^), and CO_2_ concentration (400 μmol mol ^−1^) were maintained stable. Assessed parameters were net photosynthetic rate (P_n_), transpiration rate (T_r_), intercellular CO_2_ concentration (C_i_), and stomatal conductance (G_s_). Photosynthetic parameters were assessed using six biological replicates per treatment (n = 6). For technical replication, a minimum of four leaves sharing the same nodal position and developmental stage were measured per biological replicate.

The fast chlorophyll a fluorescence transient (OJIP) and 820-nm modulated reflection (MR) were analyzed using an M-PEA analyzer (Hansatech, Norfolk, UK), following the established protocol described by Strasser et al. [96]. To ensure complete oxidation of reaction centers, leaf samples were subjected to a 30-min dark adaptation period prior to measurement. The OJIP transient was elicited by a saturating red light pulse (3000 μmol m^−2^ s^−1^), while the MR signal, indicative of the plastocyanin and P700 redox state in PSI, was monitored under far-red illumination (250 μmol m^−2^ s^−1^). JIP-test parameters were calculated from the fluorescence kinetics (Table 5). Based on the definition provided by Li et al. [97], the normalized relative variable fluorescence (Vt) at time t was calculated using the formula: V_t_ = (F_t_ − F_0_)/(F_m_ − F_0_). The difference in Vt between EBR-treated and control plants (ΔV_t_) was computed: ΔVt = Vt _(EBR treatment)_ − Vt _(control)_. PSI maximal redox activity (ΔI/I_0_) and PSII-PSI coordination [Φ_(PSI/PSII)_] were assessed from the 820 nm MR curves using the formulas: ΔI/I_0_ = (I_0_ − I_m_)/I_m_, and Φ_(PSI/PSII)_ = (ΔI/I_0_)/ψ_0_. The chlorophyll fluorescence analysis was conducted with 10 biological replicates (n = 10) per treatment. During replicate assays, a minimum of three leaves at an identical position and developmental stage were measured concurrently.

### 4.9. Root Morphology

Root morphology of *P. vulgaris* was assessed through digital scanning using an Epson Expression 12000 XL root scanner (Seiko Epson Co., Ltd., Tokyo, Japan), with data processed by the WinRHIZO root analysis system (Regent Instruments, Sainte Foy, QC, Canada). For measurement, root systems were carefully extracted from each treatment group and gently washed in deionized water to remove adhered soil without compromising fine root structure, and then flattened to minimize root overlap. The root system assay included 15 biological replicates per treatment, which were derived from 5 pots with 3 plants each (n = 15). Each sample was measured with three technical replicates.

### 4.10. Plant Morphology and Biomass Measurement

The count of lateral branches and inflorescences per plant was meticulously recorded. Stem length and spica length were precisely measured utilizing a calibrated ruler and a vernier caliper, respectively. Following desiccation to achieve constant mass, biomass accumulation (dry weight) was quantified using an electronic balance (FA1204B; Jingke Tianmei, Shanghai, China). The growth parameters of *P. vulgaris* were measured from a total of 12 plants (n = 12), with three replicate measurements taken per plant.

### 4.11. Determination of Total Phenols and Secondary Metabolites

Total phenolic content was quantified spectrophotometrically following the protocol established by Susamcı et al. [98]. Briefly, 0.2 g of dried spica powder was subjected to extraction using 10 mL of 80% methanol within a 50 °C ultrasonic bath for a duration of 30 min. Subsequently, 0.5 mL of the extract was reacted with 2.5 mL of 10% Folin–Ciocalteu reagent and 2 mL of 7.5% sodium carbonate. After incubation in darkness at room temperature for 60 min, absorbance was measured at 765 nm. The total phenolic content was quantified using a gallic acid standard curve and expressed as mg·g^−1^ DW. The total phenolic content (TPC) of spicas harvested from three individual potted *P. vulgaris* plants (biological replicates, n = 3) was determined. Each sample was analyzed in triplicate.

The contents of four active compounds, including caffeic acid, rosmarinic acid, ferulic acid, and hyperoside, were quantified using a modified HPLC method [19,99]. Dried spica powder (0.2 g) was extracted with 20 mL of 80% methanol containing 1% formic acid via ultrasonication at 50 °C for 30 min. Following centrifugation, the clarified supernatant was filtered and subsequently analyzed on an Agilent 1260 HPLC system (Agilent Technologies, Santa Clara, CA, USA). The system was configured with a Waters C18 column (250 mm × 4.6 mm) and a Diode Array Detector. Chromatographic separation was achieved using a gradient elution program with methanol and 0.2% NaH_2_PO_4_, at a volumetric flow rate of 0.8 mL min^−1^, a column temperature of 30 °C, and a detection wavelength of 325 nm. Compound identification was achieved by comparing retention times and spectral profiles with those of authenticated reference standards. Quantification was conducted employing the external standard method, with results expressed as mg per gram of dry weight (mg g^−1^ DW). Chromatographically pure standards (caffeic acid, ferulic acid, rosmarinic acid, hyperoside; Shanghai Yuanye Biotech Co., Ltd., Shanghai, China) were used to generate standard curves according to the established method [100]. The secondary metabolite content in spicas harvested from three independent potted plants of *P. vulgaris* (n = 3) was determined. Each sample was assayed in three technical replicates.

### 4.12. Gene Expression Analysis

Transcript abundance of four pivotal genes in the phenylpropanoid–tyrosine branch—cinnamate-4-hydroxylase (*PvC4H*), 4-coumarate-CoA ligase (*Pv4CL*), phenylalanine ammonia-lyase (*PvPAL*), and tyrosine aminotransferase (*PvTAT*)—was absolutely quantified by real-time quantitative PCR. Total leaf RNA was isolated with RNAiso Plus (TIANGEN Biotech, Beijing), treated with DNase I, and reverse-transcribed using the M5 Super-qPCR RT Kit (GENEWIZ, Suzhou, China). Primer pairs were either computationally designed for *Pv4CL* (Primer-BLAST) or adopted from [99] (Table 6). Amplifications were performed in 10-μL reactions containing 1 × SYBR Green Supermix on a Bio-Rad CFX96 cycler [99]. Relative expression was normalized to β-actin and calculated via the 2^−ΔΔCt^ method; gene expression levels were analyzed from three individual potted *P. vulgaris* plants (biological replicates, n = 3) and reported as mean ± SE. Each sample was measured with three technical replicates.

### 4.13. Statistical Analysis

All data met the assumptions of normality (Shapiro–Wilk test) and homogeneity of variance (Levene’s test) before ANOVA. One-way ANOVA, followed by Duncan’s multiple range test (*p* < 0.05), was employed to assess statistical differences among groups using SPSS 13.0. (SPSS Inc., Chicago, IL, USA). Data are presented as mean (±SD), derived from at least three independent biological replicates per biochemical measurement.

## 5. Conclusions

This study confirms that drought stress significantly inhibits the growth and metabolism of *P. vulgaris*, characterized by intensified lipid peroxidation, decreased photosynthetic efficiency, and reduced biomass. Foliar application of exogenous 24-epibrassinolide (EBR) significantly enhances the drought resistance, yield, and quality of *P. vulgaris* by strengthening the antioxidant system, optimizing photosynthetic physiology, promoting root and shoot growth, and activating the phenylpropanoid and tyrosine metabolic pathways. Among the tested concentrations, 0.1 µmol L^−1^ EBR exhibits the optimal effect, while the concentration range of 0.05–0.1 µmol L^−1^ also yields significant improvements, indicating this interval as the optimal application range. Therefore, foliar EBR application represents a highly promising strategy for high-yield and high-quality cultivation of *P. vulgaris* in arid and drought-prone regions, such as central and northwestern China, with considerable potential for widespread adoption. This study offers a new perspective, indicating that EBR can not only mitigate the negative effects of drought but also enhance both yield and medicinal quality.

## Figures and Tables

**Table 1:** Response of energy flux in drought-stressed *P. vulgaris* leaves to different EBR concentrations (Mean ± SD, n = 10, *p* < 0.05).

Parameter	CK	DR	EBR0.01	EBR0.05	EBR0.1	EBR0.2
ABS/RC	1.05 ± 0.04 e	1.51 ± 0.07 a	1.32 ± 0.03 bc	1.22 ± 0.08 cd	1.16 ± 0.03 d	1.34 ± 0.1 b
DI_0_/RC	0.16 ± 0.01 d	0.35 ± 0.03 a	0.28 ± 0.03 b	0.24 ± 0.02 bc	0.22 ± 0.003 c	0.29 ± 0.04 b
Tr_0_/RC	0.89 ± 0.02 d	1.15 ± 0.04 a	1.05 ± 0.02 b	0.98 ± 0.06 bc	0.94 ± 0.03 cd	1.05 ± 0.05 b
ET_0_/RC	0.55 ± 0.01 b	0.66 ± 0.06 a	0.63 ± 0.02 ab	0.60 ± 0.05 ab	0.58 ± 0.05 ab	0.63 ± 0.05 ab
RC/CS_m_	26,792.95 ± 1092.63 a	13,806.68 ± 1040.97 d	16,366.11 ± 1285.26 c	17,900.29 ± 620.56 bc	18,868.83 ± 1040.11 b	16,214.68 ± 1810.20 c
φD_0_	0.155 ± 0.007 d	0.234 ± 0.007 a	0.208 ± 0.019 b	0.198 ± 0.01 bc	0.187 ± 0.006 c	0.214 ± 0.018 ab
ABS/CS_m_	33,554.50 ± 614.48 a	24,888.50 ± 1184.02 b	25,992.50 ± 1597.54 b	26,102.50 ± 495.59 b	26,606.67 ± 513.84 b	25,922.50 ± 1030.25 b
DI_0_/CS_m_	5211.50 ± 321.73 b	5823.00 ± 106.39 a	5371.25 ± 216.57 b	5160.25 ± 324.27 b	4978.67 ± 158.19 b	5192.00 ± 239.00 b
Tr_0_/CS_m_	28,343.00 ± 292.74 a	19,065.50 ± 1084.32 c	20,621.25 ± 1768.44 bc	20,942.25 ± 370.42 bc	21,628.00 ± 152.42 b	19,230.50 ± 2912.57 bc
ET_0_/CS_m_	17,598.00 ± 296.98 a	10,903.25 ± 461.48 d	12,285.50 ± 684.25 bc	12,784.50 ± 600.07 b	13,199.33 ± 764.41 b	11,433.50 ± 1412.09 cd

Different lowercase letters are used to denote significant differences among treatments.

**Table 2:** Effects of different concentrations of EBR on the root architecture of *P. vulgaris* under drought stress (Mean ± SD, n = 15, *p* < 0.05).

Treatment	Root Length (cm)	Root Surface Area (cm^2^)	Root Volume (cm^3^)	The Number of Root Tip	Branch Number
CK	2427.68 ± 65.75 a	372.44 ± 8.52 a	4.55 ± 0.09 a	3991.67 ± 87.56 a	2266.67 ± 97.45 a
DR	1170.32 ± 50.18 f	171.69 ± 8.53 f	2.01 ± 0.13 e	2694.00 ± 302.23 d	750.60 ± 48.40 e
EBR0.01	1665.44 ± 28.74 d	253.65 ± 5.62 d	3.08 ± 0.18 d	3219.75 ± 324.79 bc	1094.25 ± 19.87 d
EBR0.05	1853.48 ± 115.18 c	287.75 ± 16.71 c	3.58 ± 0.15 c	3433.00 ± 360.62 b	1555.33 ± 229.52 bc
EBR0.1	2013.44 ± 139.33 b	314.79 ± 6.02 b	3.92 ± 0.15 b	3655.67 ± 142.66 ab	1740.33 ± 183.56 b
EBR0.2	1544.69 ± 37.06 e	239.08 ± 20.52 e	2.93 ± 0.42 d	3038.33 ± 325.59 cd	1376.33 ± 180.03 c

Different lowercase letters are used to denote significant differences among treatments.

**Table 3:** Effects of exogenous EBR on the growth traits of *P. vulgaris* under drought stress. (Mean ± SD, n = 3, *p* < 0.05).

Treatment	Stem Length (cm)	Spica Length (cm)	Number of Branches per Plant(cm)	Number of Spica per Plant	The Weight of Spica per Plant (g)	The Total Weight per Plant (g)
CK	14.77 ± 0.93 a	3.89 ± 0.26 a	6.63 ± 0.52 a	11.88 ± 0.64 a	0.64 ± 0.02 a	5.52 ± 0.15 a
DR	12.63 ± 0.34 c	2.67 ± 0.25 c	3.88 ± 0.64 d	7.25 ± 0.89 d	0.40 ± 0.03 e	3.61 ± 0.16 e
EBR0.01	13.76 ± 0.62 b	3.04 ± 0.20 b	4.50 ± 0.53 bc	8.38 ± 0.52 c	0.44 ± 0.03 d	4.08 ± 0.09 d
EBR0.05	14.18 ± 0.69 ab	3.09 ± 0.24 b	4.75 ± 0.46 b	8.88 ± 0.83 bc	0.48 ± 0.02 c	4.80 ± 0.12 c
EBR0.1	14.27 ± 0.61 ab	3.22 ± 0.15 b	4.88 ± 0.64 b	9.38 ± 0.92 b	0.54 ± 0.03 b	5.13 ± 0.20 b
EBR0.2	12.98 ± 0.25 c	2.76 ± 0.24 c	4.00 ± 0.53 cd	7.50 ± 0.93 d	0.41 ± 0.04 de	3.91 ± 0.10 d

Different lowercase letters are used to denote significant differences among treatments.

**Table 4:** Effects of different concentrations of exogenous EBR on the accumulation of secondary metabolites in *P. vulgaris* under drought stress (mg g^−1^ DW, n = 3).

Treatment	Total Phenols	Caffeic Acid	Ferulic Acid	Rosmarinic Acid	Hyperoside
CK	4.467 ± 0.161 e	0.026 ± 0.003 e	0.275 ± 0.018 d	1.341 ± 0.064 e	0.120 ± 0.010 e
DR	5.384 ± 0.194 d	0.037 ± 0.005 d	0.311 ± 0.023 cd	1.606 ± 0.109 d	0.148 ± 0.011 d
EBR0.01	5.976 ± 0.309 bc	0.047 ± 0.005 c	0.443 ± 0.051 b	1.886 ± 0.134 c	0.182 ± 0.013 c
EBR0.05	6.471 ± 0.521 b	0.057 ± 0.003 b	0.572 ± 0.015 a	2.182 ± 0.062 b	0.219 ± 0.012 b
EBR0.1	7.347 ± 0.379 a	0.081 ± 0.006 a	0.623 ± 0.055 a	2.769 ± 0.146 a	0.267 ± 0.011 a
EBR0.2	5.659 ± 0.438 cd	0.032 ± 0.002 de	0.350 ± 0.019 c	1.656 ± 0.047 d	0.159 ± 0.007 d

The data are represented as the mean ± standard deviation. Significant differences are indicated by different lowercase letters above the bars according to Duncan’s multiple range test (*p* < 0.05).

**Table 5:** Parameters in JIP test analysis.

Fluorescence Parameters	Description
W_K_ = (F_K_ − F_0_)/(F_J_ − F_0_)	Normalized relative variable fluorescence
V_J_ = (F_J_ − F_0_)/(F_m_ − F_0_)	Relative variable fluorescence intensity at the J step
M_0_ = 4(F_300μs_ − F_0_)/(F_m_ − F_0_)	Initial slope of the relative variable fluorescence of the relative rate at which *Q*_A_ is reduced
φE_0_ = ET_0_/ABS = [1 − (F_0_/F_m_)] ψ_0_	Quantum yield for electron transport
ABS/RC = M_0_(1/V_J_) (1/φP_0_)	Absorption flux per reaction center
TR_0_/RC = M_0_(1/V_J_)	Trapped energy flux per RC
ET_0_/RC = M_0_ (1/V_J_) ψE_0_	Electron transport flux per RC
DI_0_/RC = (ABS/RC) − (TR_0_/RC)	Dissipated energy flux per RC
RC/CS_m_ = φP_0_ (V_J_/M_0_) (ABS/CS_m_)	Density of RCs per excited cross-section (CS)
ABS/CS_m_ ≈ F_m_	Absorbed energy flux per CS
TR_0_/CS_m_ = φP_0_(ABS/CS_m_)	Trapped energy flux per CS
ET_0_/CS_m_ = φE_0_(ABS/CS_m_)	Electron transport flux per CS
DI_0_/CS_m_ = ABS/CS_m_−TR_0_/CS_m_	Dissipated energy flux per CS
F_v_/F_m_	Maximal quantum yield of PSII photochemistry
PI_ABS_ = (RC/ABS) [φP_0_/(1 − φP_0_)] [ψ_0_/(1 − ψ_0_)]	Performance index on absorption basis
φDo	Quantum ratio for dissipated energy

**Table 6:** Primers used for qRT-PCR Analysis.

Gene	Genbank Accession Number	Primer Name	Primer Sequence(5′ → 3′)	PCR Product (bp)
*Pv4CL*	KJ010817.1	*Pv4CL* forward	CCACCATGGCCAATCCCTATT	114
		*Pv4CL* reverse	CATAGTCCCGCACCTTGTCG	
*PvC4H*	KJ010816	*PvC4H* forward	ATCGTTGTCGCCGCCGTTGTGT	136
		*PvC4H* reverse	CGTAGTCGGTGAGGTTTCGGTGGTTC	
*PvPAL*	KJ010815.1	*PvPAL* forward	TCCGTGCTTGTGTGTTTGTGCCTGTC	203
		*PvPAL* reverse	GGCTTCCTGAACTCCTCCACCATCCT	
*PvTAT*	KM053278	*PvTAT* forward	CGTCTACTCGCATCAGCATCTCAGGA	194
		*PvTAT* reverse	GCCAACCAGGGATCAACCACCTCTTC	
β-actin	KJ010818	β-actin forward	GCAGTTCTCTCCCTATACGCCAGTGG	205

## Data Availability

The original contributions presented in this study are included in the article. Further inquiries can be directed to the corresponding author.
